# Detection of antibacterial activity of lactic acid bacteria, isolated from Sumba mare’s milk, against Bacillus cereus, Staphylococcus aureus, and Escherichia coli

**DOI:** 10.5455/javar.2022.i568

**Published:** 2022-01-15

**Authors:** Maxs U. E. Sanam, Annytha I. R. Detha, Nelsi Kurniawati Rohi

**Affiliations:** 1 Laboratory of Animal Disease and Veterinary Public Health, Faculty of Veterinary Medicine, Universitas Nusa Cendana, Kupang, Indonesia; 2 Faculty of Veterinary Medicine, Universitas Nusa Cendana, Kupang, Indonesia

**Keywords:** LAB, mare’s milk, food biopreservation, antibacterial agent

## Abstract

**Objective:**

The purpose of this research is to detect the antibacterial properties of lactic acid bacteria (LAB) against pathogenic bacteria.

**Materials and Methods:**

Isolation and determination of *Lactobacillus* spp. Testing of the antibacterial activity of LAB was conducted using filtrate and nonfiltrate forms. The lactic acid bacterial isolates were confirmed to be identified through Gram staining, cell shape, catalase testing, and motility testing.

**Results:**

The results of the analysis of the LAB inhibition zone using filtrate and nonfiltrate forms against the bacteria *Bacillus cereus* were included in the very strong category. The results of the analysis of the LAB inhibitory zone using filtrate and nonfiltrate forms and the agar well method against *Staphylococcus aureus* bacteria were classified into the very strong category. The results of the LAB inhibitory zone analysis using filtrate and nonfiltrate forms with the well method against *Escherichia coli* bacteria are included in the very strong category, whereas the results from the LAB inhibitory zone analysis using the filtrate and nonfiltrate forms with the agar diffusion method (disks) are included in the strong category.

**Conclusion:**

Based on the results, LAB isolated from Sumba mare’s milk displayed antibacterial activity in the strong and very strong categories against pathogenic bacteria such as *B. cereus, S. aureus,* and* E. coli.*

## Introduction

Lactic acid bacteria (LAB) have characteristics of Gram-positive bacteria, are nonsporulating, and are tolerant of anaerobes. LAB are fermentative bacteria that produce antimicrobial compounds such as lactic acid [1,2]. LAB are divided into two groups: those that produce lactic acid as the main product of glucose fermentation are called homofermenters, and those that produce the same molar amount of lactate, carbon dioxide, and ethanol from hexose are called heterofermentative [3,4]. The LAB are known to improve immune-mediated health complications such as allergies, atopic dermatitis, rhinitis, oral tolerance, cancer, and inflammatory diseases [5]. Another study showed that consumption of LAB-fermented food products can reduce cholesterol and the risk of cardiovascular disease [6]. In addition, increased consumption of LAB-fermented foods can be applied to prevent diarrhea, reduce lactose intolerance, produce conjugated linoleic acid, decompose phytic acid (an inhibitor of mineral absorption in the intestine), and increase intestinal microbial balance [5,7].

Several applied studies have been carried out to determine the function of various LAB properties, including antibacterial activity with a broad spectrum [4,8–10]. This function is due to the antimicrobial property that can inhibit the growth of other microorganisms and is promising as an alternative antibiotic for certain bacteria [2,8,11–13]. Research on LAB candidates for antibiotic studies was further developed after the emergence of antibiotic resistance problems [14–16], mainly because bacteria are responsible for numerous foodborne diseases such as Bacillus cereus, Staphylococcus aureus, and Escherichia coli. Product metabolites of LAB are important in inhibiting microbial growth. The mode of action of LAB is a synergistic collaboration between lactic acid and several other metabolites (hydrogen peroxide, diacetyl, reuterin, and bacteriocins) that produce bactericidal action against pathogenic bacteria.

Based on its habitat, LAB can isolate natural ingredients such as plants and fruits [17], wine [1], and milk [2,18,19]. Recent research explains the presence of LAB isolated from Sumba mare’s milk [20,21]. Sumba horses have specifications in terms of maintenance patterns. Sumba horses are kept in the vast savanna and are released to find their food according to their taste. This impacts the types of feed obtained in nature, which are very diverse and may cause variations in the LAB present in the milk content. From the perspective of public health, LAB can be used as an alternative treatment to treat foodborne diseases, primarily when associated with the issue of antimicrobial resistance. The purpose of this research is to detect the antibacterial property of LAB against pathogenic bacteria. The pathogenic bacteria that were challenged to detect the antimicrobial function of LAB were B. cereus, S. aureus, and E. coli.

## Materials and Methods

This research was conducted from August to September 2018 at the Animal Disease and Veterinary Public Health, Faculty of Veterinary Medicine, Universitas Nusa Cendana. The stages of this study include extracting isolates of LAB from Sumba mare’s milk and identifying the antimicrobial activity of LAB extracted from the mare’s milk against pathogenic bacteria B. cereus*,* S. aureus*,*and E. coli.

The research materials used were LAB isolated from Sumba mare’s milk, De Man, Rogosa, and Sharpe (MRS) agar, MRS broth, Mueller Hinton agar media, sulfide indole motility media, aquades, violet crystal solution, iodine solution, 96% alcohol, safranin, physiological NaCl, 70% alcohol, hydrogen peroxide, chloramphenicol antibiotic disks, blank disks, McFarland standards, bacterial cultures of B. aureus*,* S. aureus*, *and E. coli, antibiotics, oil immersion, filter paper, cotton wool, masks, gloves, and label paper.

### Method analysis

The main focus of this study was to measure the inhibitory diameter of LAB on the growth of bacteria that cause important pathogens (B. aureus, S. aureus, and E. coli). Isolation and determination of Lactobacillus spp. were carried out in accordance with LVS ISO 15214: 1998 guidelines using MRS media (Tween, OXOID, UK). Media preparation procedures are guided by the LVS CEN ISO/TS 111331:2009 guide. Similarly, the sample dilution technique is carried out according to ISO 6887-5:2010 using a salt–peptone solution. The diameter of the inhibition zone was measured after incubation of LAB for 72 h at 37°C on MRS agar media [22,23]. Confirmation of lactic acid bacterial isolates was identified through testing of Gram-stain, cell shape, catalase testing, and motility testing [24].

According to Pro-Lab Diagnostics, the detection of antimicrobial activity against pathogenic bacteria was conducted. A volume of 1 ml of active LAB suspension of Sumba mare’s milk from MRS broth was taken using a micropipette and then poured into a test tube. The test tube containing LAB was centrifuged at 3,000 rpm for 30 min to separate the liquid from the filtrate. Next, the separated solution was filtered using a 0.45-µm millipore membrane. The testing of the effectiveness of the filtrate of LAB against pathogenic bacteria was based on several research studies [25,22]. The testing of the antimicrobial activity of LAB bacteria using filtrate and nonfiltrate forms was carried out based on previous research. This test was conducted using the disk diffusion method and the agar well method. The pathogenic bacteria were B. cereus, S. aureus, and E. coli. LAB from Sumba mare’s milk was used as an antimicrobial. Also, a positive control using an antibiotic (chloramphenicol), and a negative control used disk blank to validate the test procedure. An antimicrobial activity test was carried out with three repetitions. The results obtained from the literature were regarding the four categories of inhibition of active compounds in bacteria. The diameter of the inhibition zone was classified as weak (5 mm), moderate (5–10 mm), strong (10–20 mm), and very strong (20–30 mm) [21,26].

## Results and Discussion

The bacteria cultures were observed at 48 h, with the following inhibitory zone results; 29.63 mm in pathogenic bacteria B. cereus, 32.03 mm in pathogenic bacteria S. aureus, and 31.23 mm in pathogenic bacteria E. coli. Inhibitory zones for antibiotics that formed within 48 h were included in the sensitive category. This is consistent with accepted standards for the diameter of a bacterial inhibitory zone, which state that chloramphenicol is resistant if the diameter of the bacterial growth inhibition produced is smaller than 20 mm, or sensitive if the resulting inhibitory diameter is greater than 21 mm [27,28].

The results of the antibacterial activity test for LAB bacteria against B. cereus using filtrate form and the agar diffusion method were observed at 48 h, with an average inhibition zone of 20.5 mm, while nonfiltrate forms did not form inhibitory zones. Furthermore, the testing of the antibacterial activity of LAB in filtrate form was carried out using wells that were observed at 48 h with an average inhibition zone of 24.7 mm, while the nonfiltrate form had an inhibition zone of 20.4 mm. The results of the inhibition zone associated with LAB using filtrate and nonfiltrate forms against the bacteria B. cereus are included in the very strong category [26].

The antibacterial activity of LAB against S. aureus bacteria in the filtrate form using the agar diffusion method (disks) was observed at 48 h with an average inhibition zone of 24.9 mm, whereas the nonfiltrate test using agar diffusion (disks) had an average inhibition zone of 13.76 mm. The testing of the antibacterial activity of LAB in the filtrate form was carried out using a well and was observed at 48 h with an average inhibition zone of 31.10 mm, while the nonfiltrate had an average inhibition zone of 21.85 mm. Given these results, the LAB inhibitory zone for filtrate and nonfiltrate forms with the agar well method against S. aureus bacteria is included in the very strong category, whereas the nonfiltrate LAB inhibitory zone using the agar diffusion method (disks) is included in the strong category.

The results of the antibacterial activity of LAB against E. coli suspension using the agar diffusion method (disks) were observed at 48 h with an average inhibition zone of 13.3 mm, while nonfiltrate was 11.65 mm. Testing the antibacterial activity of LAB in Sumba mare’s milk isolates using well agar was observed at 48 h with an average inhibition zone of 22.3 mm, while nonfiltrate was 22.1 mm. Based on the LAB inhibitory zone results obtained by the filtrate and nonfiltrate forms, using the well method against E. coli bacteria is included in the very strong category, whereas the LAB inhibitory zone obtained by the filtrate and nonfiltrate forms using agar diffusion method (disks) is included in the strong category.

**Figure 1.**
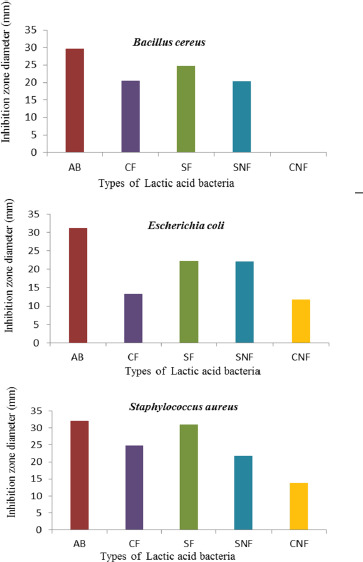
Inhibitory zone diameter of LAB taken from Sumba mare’s milk isolates against *B. cereus, E. coli*, and *S. aureus.* AB: Chloramphenicol; CF: LAB in the form of disk filtrate; CNF: nonlactic acid filtrate bacteria; SNF: Non-LAB wells filtrate; SF: LAB filtrate wells.

The identification of the antibacterial activity of LAB, isolated from Sumba mare’s milk, was conducted *in vitro* against pathogenic bacteria (B. cereus, S. aureus, and E. coli)(Fig. 1). After incubation for 24–48 h, the test results showed an inhibition zone marked by the appearance of a clear zone around the LAB against the pathogenic bacteria B. cereus, S. aureus, and E. coli.

The results from this research show that the inhibition zone associated with LAB using filtrate and nonfiltrate forms against B. cereus, S. aureus, and E. coli is included in the very strong category. Several previous studies mentioned that LAB had an antimicrobial ability because it produced a variety of antimicrobial compounds [29]. Some important compounds, including organic acids, diacetyl, hydrogen peroxide, bacteriocin, and reuterin, have antimicrobial power with a variety of characteristics, such as specific or nonspecific molecular weights. Each of these compounds has a different antimicrobial object-level [4].

As one of the metabolites of the LAB process, organic acids have a level of antimicrobial activity within a broader spectrum than other compounds. This broad-spectrum is known to be a result of a synergistic collaboration between lactic acid and acetic acid that produces a bactericidal for pathogenic bacteria such as Salmonella typhimurium [3,9,12]. Moreover, the effect of lactic acid causes a change in pH level, which leads to low acidity. This adds to the antimicrobial properties of organic acids, including bacteria, fungi, and yeast [4]. Hydrogen peroxide is another compound produced by LAB. This compound is produced when there is nicotinamide adenine dinucleotide oxidase and superoxide dismutase activity. LAB will produce catalase if heme is not available. In contrast, when the catalase is not produced by LAB, there will be an accumulation of antimicrobial peroxide through a strongly produced oxidation effect [30]. The strength of the peroxide is further strengthened when additional lactoperoxidase and thiocyanate are found in natural products, especially milk [31–33]. Peroxidase is known to have the ability to inhibit the growth of Lactococcus and Gram-negative Pseudomonas ssp, which are often associated with an incidence of foodborne diseases [15,16].

In addition, LAB produces 5-carboxylic acid pyrrolidone. This compound is known to have the ability to kill bacteria such as B. subtilis and Enterobacter cloacae. However, this compound is only produced by Lactobacillus casei *ssp.* casei and L. casei *ssp.* Pseudoplantarum [34]. Diacetyl compounds are produced by LAB in the process of transformation of citrate through pyruvate. Diacetyl produces a characteristic odor of butter and can reduce the pH level [15,35]. Other studies explain that diacetyl has been shown to be effective in inhibiting pathogenic bacteria, such as the genera Salmonella, Yersinia, Escherichia, Aeromonas, and Bacillus .

One of the compounds produced by LAB is bacteriocin. Bacteriocin is a protein compound excreted by bacteria that inhibits bacterial growth by destroying the bacterial cells, causing development disruption [37]. Another antimicrobial compound that LAB produces is Reuterin, which has the ability to bind to a group of SH enzymes, such as ribonucleotide reductase [38]. It is known as a compound produced during anaerobic conditions by L. reuteri*, *L. brevis*,* L. buchneri*,* L. collinoides*,*and L. corniformis [15]. Reuterin antibacterial compounds in LAB are known to have the ability to inhibit the growth of a number of enterobacteria such as Salmonella sp and Shigella sp, genus Clostridium, Staphylococcus [39], Listeria [40], and yeast of the genus Candida [41].

The antibacterial properties of LAB promote the frequent use of these natural ingredients as biopreservation for food [19,41–43]. The results of this study further confirm that the antimicrobial nature of LAB can be utilized for several functions, including as an alternative antibiotic [2,13], especially to overcome the problem of antibiotic resistance [16,44], anti-fungus [41,45], food bio-preservatives [46], and to help increase the absorption of calcium and magnesium [17]. Further research needs to examine the characteristics of DNA sequencing in LAB in order to be able to specifically identify the species of LAB isolated from mare’s milk.

## Conclusion

Based on the test results, LAB isolates taken from Sumba mare’s milk contain compounds that are in the strong and very strong categories of antimicrobial activity against *B. cereus*, *S. aureus*, and* E. coli.*

## List of Abbreviations

LAB, Lactic acid bacteria; DNA, Deoxyribonucleic acid;
